# The Dynamic Genetic-Hormonal Regulatory Network Controlling the Trichome Development in Leaves

**DOI:** 10.3390/plants8080253

**Published:** 2019-07-28

**Authors:** Marco Fambrini, Claudio Pugliesi

**Affiliations:** Department of Agriculture, Food and Environment (DAFE), University of Pisa, Via del Borghetto, 80-56124 Pisa, Italy

**Keywords:** trichomes, transcription factors, hormones, endoreduplication cycle, epigenetic mechanisms

## Abstract

Plant trichomes are outgrowths developed from an epidermal pavement cells of leaves and other organs. Trichomes (also called ‘hairs’) play well-recognized roles in defense against insect herbivores, forming a physical barrier that obstructs insect movement and mediating chemical defenses. In addition, trichomes can act as a mechanosensory switch, transducing mechanical stimuli (e.g., insect movement) into physiological signals, helping the plant to respond to insect attacks. Hairs can also modulate plant responses to abiotic stresses, such as water loss, an excess of light and temperature, and reflect light to protect plants against UV radiation. The structure of trichomes is species-specific and this trait is generally related to their function. These outgrowths are easily analyzed and their origin represents an outstanding subject to study epidermal cell fate and patterning in plant organs. In leaves, the developmental control of the trichomatous complement has highlighted a regulatory network based on four fundamental elements: (i) genes that activate and/or modify the normal cell cycle of epidermal pavement cells (i.e., endoreduplication cycles); (ii) transcription factors that create an activator/repressor complex with a central role in determining cell fate, initiation, and differentiation of an epidermal cell in trichomes; (iii) evidence that underlines the interplay of the aforesaid complex with different classes of phytohormones; (iv) epigenetic mechanisms involved in trichome development. Here, we reviewed the role of genes in the development of trichomes, as well as the interaction between genes and hormones. Furthermore, we reported basic studies about the regulation of the cell cycle and the complexity of trichomes. Finally, this review focused on the epigenetic factors involved in the initiation and development of hairs, mainly on leaves.

## 1. Introduction

The epidermis is the superficial coating layer that wraps leaves and the primary body of the stem and it is in direct contact with the atmosphere, and is therefore a protective barrier against abiotic and biotic factors. The epidermis is not a homogeneous tissue: it is made up of epidermal cells and by annexed (or specialized) cells, such as stomata and trichomes or hairs [1,2,3,4,5,6,7].

Trichomes are epidermal projections consisting of single or groups of cells with different shapes, sizes, structures, and functions. Located on the surface of any part of the plant body, they can be persistent or ephemeral, alive or dead. Trichomes can be unicellular, multicellular, simple or branched, starry, squamiform, or glandular [1,3,7,8]. Every single hair originates from an epidermal pavement cell (initial cell). In some cases, the latter forms, by distension, a long extroflexion, generating a single-cell hair. In contrast, multicellular trichomes develop when the mother cell undergoes repeated divisions. Often, there are several types of hairs in the same organism, and the term “trichome complement” is referred to as the set of all the hairs present on the surface of a plant, with different characteristics and functions [5,9]. The protective function of the hair is very common. Hairs with this role are generally dead and full of air that gives brightness and a whitish color, a sign of the reflection of the light of which they are capable; in this way, they ensure an effective protection against solar radiation and preserve the plant (especially the leaves) from excessive water loss through transpiration. More rarely, live trichomes covered only by a thin cuticle are thought to enable at least some transpiration of plants growing in extremely humid environments. Hairs appear to enhance this process by increasing the surface area on which water droplets can accumulate. Trichomes play a role in plant defense against many insect herbivores, both by forming a physical barrier that obstructs insect movement and by acting as a mechanosensory switch, transducing mechanical stimuli into physiological signals [10,11]. In *Arabidopsis*, when the trichomes are mechanically stimulated by insect movement, a buckling deformation is produced on trichome base regions [11]. Such buckling instability can elicit cytosolic Ca^2+^ fluctuations and apoplastic pH shifts in surrounding cells, transducing mechanical signals to epidermal pavement cells [11]. The buckling instability and a calcium oscillation are linked to a non-uniform spatial distribution of cell wall mechanical properties, from branch tips to the base of trichomes [10]. Zhou et al. [11] suggested that these physiological responses enhance the production of chemical deterrents above the constitutive levels and their storage, as well as facilitating release. Indeed, trichomes act as a sensory switch for perceiving touch by potential insect attacks [11].

In addition to their protective role, other functions of trichomes include those of support, absorption, secretion, dissemination, and perception of external stimuli [8,9]. Notably, glandular trichomes are metabolically highly diverse, and they synthesize, store, and release a large number of compounds not directly involved in the normal growth and development of the trichomes, such as isoprenoids, flavonoids and phenylpropanoid, alkaloid, *O*-acyl sugars, and defensive proteins [12,13,14,15,16,17]. Many of these chemicals are thought to function in defense against herbivores and arthropods, and they are also important molecules in human health [18,19,20,21,22]. Hairs are useful outgrowths against biotic stresses but are also involved in plant defense towards several abiotic stresses [23,24,25,26]. Several plants accumulate UV-absorbing compounds, such as flavonols, in trichomes, which further protect the underlying photosynthetic tissues from damaging amounts of UV-A and UV-B radiations [27]. There is also evidence that trichomes are structural adaptations to low temperature and ozone [28], as well to heavy metals in contaminated soil [26,29]. Peculiar structures are the glandular type trichomes described in Orobanchaceae, because they can help in the gaining of nutrients from the xylem of parasitized plants [30]. Moreover, in the *Gossypium* genus, they are essential in the coating of the ovule for seed dispersion but also for manufacture of clothing fabrics [31].

The presence or absence of trichomes identifies specific traits in the heterochronic processes mainly investigated in *Arabidopsis* [32,33,34]. In this species, mature hairs are present on leaves, stems, and sepals. Usually, plant embryos are devoid of trichomes and, at the seedling stage, cotyledons and hypocotyls of *Arabidopsis* are glabrous; the first trichomes differentiate on the adaxial surface of the first pair of leaves [35,36]. Trichome precursor cells become visible in leaf primordia of 100 μm in length [1]. Trichome development starts near the distal end of the maturing leaf and proceeds basipetally. The initiation of trichomes is regularly spaced and each belongs to a different cell lineage [36]. The pattern of initiation is influenced by a field of inhibition originating within each developing trichome and extends two–three cells beyond the hair [1,36]. However, the establishment of the trichome pattern in vivo is not an obvious phenomenon [8,37]. In *Arabidopsis*, the epidermal cells destined to become trichomes cease to divide, to enter in endoreduplication cycles in which DNA replication continues in the absence of nuclear and cellular division. On mature leaves, trichomes are branched with a single stalk sustaining three spikes [35].

In *Begonia dregei*, leaf shape and trichomatous complements are linked to morphological traits [38]. Furthermore, in hybrid plants, leaves characterized by deep incisions show a high density of elongated trichomes [38]. *Arabidopsis* mutations for leaf shape can also affect the trichome branching pattern [1]. Finally, irregular patterns of trichome differentiation are detected in specific leaf areas, as well as in galls induced by insect colonization [39].

Here, we focus on the molecular mechanisms in unicellular and multicellular trichome formation, and the regulation of the cell cycle in its initiation and morphogenesis. Furthermore, we discuss the influence of phytohormones and their interactions on gene expression, affecting trichome initiation and development. Finally, the epigenetic factors involved in trichome morphogenesis are briefly summarized.

## 2. Genes Involved in the Initiation and Growth of Trichomes Overall in Model Species

Trichome development in *Arabidopsis thaliana* has become a well-studied model system because of the availability of several mutants with defects in the initiation and development of these structures. In this species, trichomes have a typical unicellular structure and their origin from the epidermis comprises three successive phases: determination of cell fate, specification, and morphogenesis [1,8,9,40]. While the other cells belonging to the epidermis continue to divide, the trichomatous cells enter in a phase characterized by one to four cycles of endoreduplication, reaching a mean DNA (C) content equal to 32C (see complexity of trichomes). The origin of the trichomes begins from epidermal pavement cells, at a stage where all the cells are predictably competent to initiate trichome differentiation. Nevertheless, the epidermal cells that generate the leaf hairs are arranged at regular intervals of distance from each other [8]. Usually, in wild type *Arabidopsis*, no trichome clusters are detected on the leaf epidermis. This requires a mechanism able to regulate the spatial arrangement of the hairs on the leaf surface [8,37]. A similar event is common in the stomata development [6].

In *Arabidopsis*, more than 70 genes are involved in the initiation and differentiation of trichomes [5,7,8,9,40]. The beginning of the epidermis trichomes consists of a regulatory network involving the activity of genes, generally divided into two basic classes: positive and negative regulators [2,8,9,41]. In *Arabidopsis*, positive regulators include several groups of transcription factors (TFs), namely, the MYB, bHLH, WDR, and C2H2 zinc finger proteins. *GLABROUS1* (*GL1*) and its paralagous *MYB23* encode R2R3-MYB TFs belonging to sub-group 15 [42]. These two proteins are functionally equivalent during trichome initiation but not during trichome branching. The *GLABRA3* (*GL3*) gene encoding a basic helix–loop–helix (bHLH) TF and the homologous *ENHANCER OF GLABRA3* (*EGL3*) gene, members of subgroup IIIf, are involved in trichome development in a partially redundant manner [43,44,45,46,47]. The nuclei in *gl3-1* mutants undergo three, rather than four, rounds of endoreduplication cycles, and this correlates with the reduced trichome branching of this mutant. The *EGL3* gene has a moderate effect on trichome number. However, *gl3*; *egl3* double mutants have a glabrous phenotype [5]. An activator is also the *TRANSPARENT TESTA GLABRA1* (*TTG1*) gene that encodes a protein containing a WD40 repeat, a highly conserved motif of about 40–43 amino acids, often ending in the Trp-Asp (W-D) residues. WD40 repeat proteins are involved in the regulation of a number of processes, including cell cycle, cell fate determination, and cell signaling [48,49,50]. *GL1* and *TTG1* appear to play a key function for trichome initiation, since the *gl1* and *ttg1* mutants exhibit almost a hairless phenotype, and in *gl1*, the development of only a few trichomes starts at the leaf margins [1,2,44,51,52]. Both *GL1* and *TTG1* control the same process of trichome development [51,52], although *TTG1* also appears to be involved in the regulation of flavonoid biosynthesis [50]. The anthocyanins do not accumulate in the *ttg1* mutant even when the plants are subjected to stress, a condition that generally determines reddish colors in the stem and leaves of wild type plants [53]. In *ttg1* mutants, the dense brown tannin produced by the inner layer of the seed coating is absent [54]. A model based on the activation of epidermal cells to differentiate trichomes involves the depletion of *TTG1*. This model hypothesizes that initially all epidermal cells express *TTG1* equally, but its level of expression decreases dramatically in cells adjacent to those that would start trichome development [5,8,9]. Really, *TTG1* can move freely between young tissues and accumulate in cells containing high levels of *GL3*; therefore, cells with a high content of the GL3/TTG1 complex will be able to develop trichomes, unlike neighboring cells, in which TTG1 will be insufficient for the competence of the epidermal cell to differentiate leaf follicles [5,8]. Supporting this depletion model, Balkunde et al. [47] showed that GL3 controls TTG1 movement, and interaction between GL3 and TTG1 is necessary for intracellular movement and epidermal distribution.

**Figure 1:**
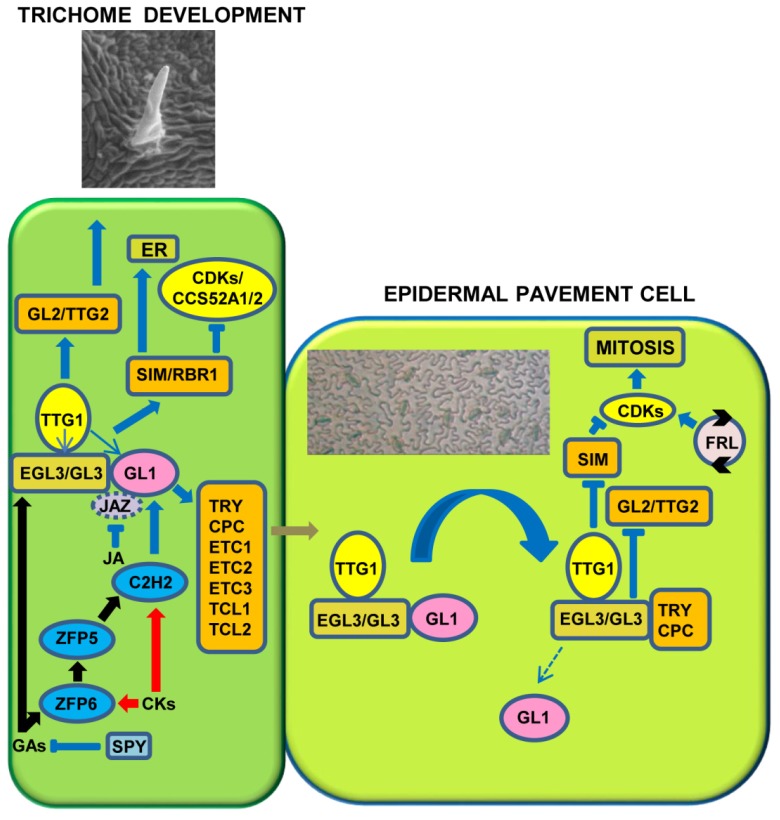
A simplified model for the acquisition of the competence of epidermal pavement cells to become trichomes in the model species *Arabidopsis thaliana*. In epidermal pavement cells, GLABRA3 (GL3) acts together with GLABRA1 (GL1) and TRANSPARENT TESTA GLABRA1 (TTG1), creating a trimeric MYB/bHLH/WD (MBW) activator complex. *TTG1* works upstream of *GL3* and *GL1*, activating their expression (thin blue arrows). Gibberellins (GAs), cytokinins (CKs), and jasmonic acid (JA) contribute positively to the regulation of trichome development. GAs activate the transcription of the *ZINC FINGER PROTEIN 6* (*ZFP6*) gene, a member of the large *C2H2* regulatory gene family (black arrow). *ZFP6* induces the expression of *ZFP5*, and then *ZFP5* promotes *GLABROUS INFLORESCENCE STEMS* (*GIS*) (*C2H2*), *GIS2*, and *ZFP8* expression (black arrows). At the same time, CKs promote *ZP6*, *ZFP8*, and *GIS2* expression (red arrows). *C2H2* members (i.e., *GIS2* and *ZFP8*) increase the transcription of *GL1* (blue arrow). The transcription levels of *GL3*, *TTG1*, and *TRY* are regulated by GAs (in the figure a single black arrow indicates the activation of the MBW complex). The *SPINDLY* (*SPY*) gene inhibits the GA signal (blue inhibitory line). JA regulates the formation of trichomes, favoring the degradation of proteins of the Jasmonate ZIM-domain (JAZ) (blue inhibitory line and blue dotted line); therefore, this hormone inhibits the interaction between JAZ with GL1 and EGL3/GL3 transcription factors (TFs). The MBW complex stimulates the development of trichomes, switching the transcription of several targets (represented by orange boxes): (i) *SIAMESE* (*SIM*) and *RETINOBLASTOMA RELATED1* (*RBR1*) as key genes in cell cycle regulation (blue arrow); (ii) GL2 and TTG2 as positive regulators (blue arrow); and (iii) R3-MYB TFs (CAPRICE (CPC), TRYPTICON (TRY), ENHANCER OF TRY AND CPC1 (ETC1), ETC2, ETC3, TRICHOMELESS1 (TCL1) and TCL2) as repressors of trichome initiation and growth (blue arrow). *SIM* and *RBR1* promote the trichome initiation from epidermal pavement cells through the down-regulation of some cyclin-dependent kinases (CDKs) and the *CELL CYCLE SWITCH 52A1* (*CCS52A1*) and *CCS52A2* genes, respectively (blue inhibitory line). Therefore, the conversion of a normal mitosis into the endoreduplication cycle (ER) is promoted (blue arrow). The R3-MYB inhibitors are capable of movement in adjacent cells (grey arrow) and replace GL1 in the MBW complex (bent and dotted blue arrows) to form a repressor complex, which cannot promote *GL2* and *TTG2* expression (blue inhibitory line), thereby inhibiting trichome fate. The repressor complex also loses ability to activate the target *SIM* gene (blue inhibitory line). A complex feedback regulatory loop (FRL, pink circle with black arrowheads), including, among other inhibitors of CDKs such as KIP-RELATED PROTEINS (KRPs) and SIM, RBR1 and E2F, TFs activate (blue arrow) some CDKs allowing cells entry into the mitotic cycle (blue arrow). TFs: transcription factors.

Collectively, these observations suggest that GL1 and TTG1 cooperate with GL3/EGL3 to make an activator trimeric complex MYB/bHLH/WD (MBW) [48,49,55,56,57,58]. This regulatory “pool” activates epidermal pavement cells to initiate the differentiation of trichomatous cells (Figure 1), promoting the transcription of the positive regulators *GLABRA2* (*GL2*) and *TRANSPARENT TESTA GLABRA2* (*TTG2*) that encode for a “Homeo Domain-Leucine Zipper” (HD-Zip) and a WRKY TF, respectively [5,7,8,9,59,60,61,62,63,64,65,66].

The *gl2* mutant produces anomalous trichomes (not expanded and unbranched) analogously to *ttg2*, in which the main consequence of the mutation is to decrease or abolish branching on trichomes. These observations indicate that these genes are also fundamental to establish the trichome complexity [61]. Notably, in *Brassica napus*, four *BnaTTG2* genes rescue the phenotypes of *Arabidopsis ttg2* mutants [67]. The over-expression of *BnaA.TTG2.a.1* also enhances the number of trichomes, both in *Arabidopsis* and *B. napus*. In both species, the *BnaA.TTG2.a.1*-over-expressing plants also show an increased resistance to salt stress [67]. Moreover, *Arabidopsis* plants under salt stress and over-expressing *BnaA.TTG2.a.1* reduce the endogenous auxin content, as well as the transcription of *TRYPTOPHAN BIOSYNTHESIS 5* (*TRP5*) and *YUCCA2* (YUC2) genes. Therefore, Li et al. [67] suggested a new function for the *Bna.TTG2* genes in the adaptation of plants to salt stress and in the hormonal metabolism. In competent cells, the MBW complex also stimulates the development of trichomes, with the activation of the *SIAMESE* (*SIM*) and *RETINOBLASTOMA RELATED1* (*RBR1*) genes, key players in the endoreduplication process (Figure 1; see also further details in the fourth paragraph).

Negative regulators that are involved in trichome initiation and outgrowth consist of at least seven genes: *CAPRICE* (*CPC*), *TRIPTYCHON* (*TRY*), *ENHANCER OF TRY AND CPC1* (*ETC1*), *ETC2*, *ETC3*, *TRICHOMELESS1* (*TCL1*), and *TCL2*, each of them coding for a member of the large family of MYB TFs, single-repeat R3 [3,7,9,41,68,69,70,71]. A phylogenetic analysis identified the amino acid sequences of TRY and ETC2 in a separate cluster with respect to the CPC, TCL1, ETC1, and ETC3 TFs [72]. These negative regulators show partially redundant roles in the initiation and differentiation of both trichomes and root hairs. An over-expression of these TFs induces glabrous phenotypes, but a single R3-MYB mutation leads to different phenotypes, indicating that these genes do not have a fully redundant activity [9]. *cpc*, *etc2*, and *etc3* mutants show a greater density of trichomes, whereas the leaves of both *try* and *cpc*; *try* double mutants differentiate more clusters of trichomes. In addition, *tcl1* and *tcl2* mutant plants do not exhibit variation in the density of trichomes on leaves, but an increase of the trichomatous complement in their reproductive organs [29,41,68,69]. These results suggest that *TRY* is leading in controlling the formation of “clusters” of leaf trichomes, while *TCL1* and *TCL2* have a functionally active role in the development of trichomes in organs of the inflorescence [8,9]. Therefore, although the functions of *CPC*, *TRY*, *ETC1*, *ETC2*, and *ETC3* are partially redundant, their gene activities are prevalent in distinct spatial domains, providing actual evidence for the correlation between gene activity and trichome differentiation in specific regions of the plant [5,8,9].

The MBW complex activates the transcription of genes, encoding the negative regulators (TRY/CPC). These inhibitors can move laterally in the epidermis between neighboring cells, competing with *GL1* and interacting with *GL3/EGL3* to establish the repressor complex GL3/EGL3-CPC/TRY-TTG1 (Figure 1). When inactive, the trimeric complex becomes unable to trigger the expression of *GL2* and *TTG2* and then represses trichome initiation in adjacent cells [56,73]. In more detail, both GL1 and GL3 include a motif for DNA binding, and the alteration of these regions represses the expression of *GL2*. *TTG1* can activate the transcription of the complex *GL3*/*GL1* and this suggests that *TTG1* operates upstream of these genes (Figure 1). *GL3* and *TTG2* are able to repress its expression, while positive regulators induce the inhibitory genes *TRY*, *CPC*, *ETC1* and *ETC3*; therefore, the activation of the promoters of the *CPC* and *TRY* genes requires a direct bond with the positive regulator *GL3* [9,56,63,74,75,76]. In particular, GL3 contains three different protein–protein interaction domains: the N-terminal MYB-interacting region that interacts with GL1/CPC/TRY, the middle portion, which includes the transactivation domain interacting with TTG1, and the C-terminal bHLH and ACT-like domain, which homo/heterodimerize [44,55]. Furthermore, TTG2 binds to W-boxes in a region of the *TRY* promoter. These W-boxes are crucial for rescuing the *try* mutant phenotype [76]. TTG2, when acting alone, is not capable of triggering *TRY* transcription, but increases the activity of *TTG1* and *GL3* by establishing a protein complex [76]. In addition, *GL1* suppresses the activation of the *TRY* promoter through the positive regulators *GL3* and *TTG1*, while the suppression of the *CPC* promoter via *TTG1* is mediated by both *GL1* and *GL3* [77]. The GL3/EGL3-CPC/TRY-TTG1 complex loses the activation activity of the target *SIM* gene. A complex feedback regulatory loop, including, among other inhibitors of cyclin-dependent kinases (CDKs) (such as KIP-RELATED PROTEINS (KRPs) and SIM), RBR1 and E2F TFs, activates some CDKs to allow cells entry into the mitotic cycle. Therefore, a regulatory loop, linking to a local autonomous circuit various positive and negative regulators, controls the activation of downstream target genes and, finally, the cell competence for trichome initiation and development (Figure 1).

The dynamic regulatory network controlling trichome initiation and development involves other TFs. A positive regulator in hair development is MYB82, a member of the R2R3-MYB TF family. In *Arabidopsis* plants, the suppression of MYB82 function produces glabrous leaves [78]. Notably, overexpression of the *MYB82* genomic sequence, but not its cDNA sequence, reduces the trichome number. This result was validated by further analyses, which indicated that at least one of the two introns in *MYB82* was essential for *MYB82* regulation of trichome development [78]. In addition, an MYB-binding box was identified in the third exon of *MYB82*, which is crucial for *MYB82* function in trichome initiation because the mutation of this motif interferes with the ability of *MYB82* to complement the *gl1* phenotype [78]. This result suggests that *MYB82* is, at least partially, functionally redundant to *GL1* [78]. Protein interaction analysis also reveals that MYB82 interacts with GL3 [78]. Therefore, in *Arabidopsis*, MYB82 is likely integrated to the activator MBW complex, playing a role in the network that regulates trichome initiation and growth [79,80,81]. However, although *MYB82* is a paralogue of *GL1*, Liang et al. [78] showed that *MYB82* has evolved distinct *cis*-regulatory elements with respect to *GL1*, which directly affect *MYB82* functions.

The activity of some genes is restricted to specific stages of trichome differentiation. For example, in *Arabidopsis*, *GLASSY HAIR* (*GLH*) genes are essential for the arrangement of the surface of the papillae in an advanced phase of trichome differentiation [82]. In *glh* mutant plants, the trichomes display a translucent aspect because of unhindered light conduction. In particular, seven distinct genetic loci have been identified. Two loci matched the *TRICHOME BIREFRINGENCE* (*TBR*) and *NOECK* (*NOK*) genes [83,84,85]. *NOK* is an *MYB* gene of the *MIXTA* subfamily [86], which in *Arabidopsis* represses branching in trichomes [83]. Both the trichomes of *glh2* and *glh4* mutant plants show a noticeable decrease in the cellulose content. The *glh6* mutant, in addition to showing a glassy trichome phenotype, displays noteworthy defects in leaf cuticular wax [82]. Lastly, *glh1* and *glh3* mutant plants produce trichomes with a severe reduction of papillae development. Therefore, both *GLH1* and *GLH3* genes could have specific key roles in the formation of trichome papillae, while *GLH2*, *GLH4*, and *GLH6* genes are likely required to add supplementary cell wall constituents [82]. *TBR* belongs to the *TRICHOME BIREFRINGENCE-Like* (*TBL*) gene family. Members of the TBL protein family influence the resistance to pathogens, tolerance to freezing, and synthesis of cellulose on the secondary wall [84]. In trichome differentiation, the gene *TBR* has a fundamental function in the cellulose content, but additionally regulates the density of trichomes on the epidermal surface [84,85].

In plants, non-specific lipid transfer proteins (nsLTPs) are part of a large multigenic family that presides over many important complex metabolic functions, such as the stabilization of cell membranes, structural organization of cell walls, and signal transduction [87]. NsLTPs regulate crucial aspects in the resistance to biotic and abiotic stress, as well as multiple phases of plant development, which include proper seed maturation, germination, and sexual reproduction [87]. Notably, in leaves of *Brassica napus*, the over-expression of *BraLTP2* causes a significant increase in the density of the trichomes but also a rise in the production of secondary metabolites [88]. Several tobacco LTPs, specifically accumulated in trichomes, have been identified [89]. *NtLTP1*, transcriptionally active in the long secretory glandular trichomes of tobacco plants, has a key function both in the lipid secretion of trichomes and in the resistance to aphid attacks [90].

Several genes, which extend the network involving repressors and activators of epidermal cell fate, trichome initiation, and differentiation, have been discovered and several examples can be depicted. The AtMYC1 bHLH TF of *Arabidopsis* was identified as a direct target of both *GL1* and *GL3* genes [91]. *AtMYC1* operates as an activator of trichome initiation, since trichome density is decreased in *atmyc1* mutants in comparison to wild type plants [91,92]. *GL3*/*EGL3* is able to substitute the activity of *AtMYC1*, while the phenotype of both *gl3* and *egl3* mutants cannot be recovered from *AtMYC1* activity, suggesting both a partially redundant role and a different function of these genes [92]. Gene expression analyses also show that *AtMYC1* operates upstream of *GL2* [92]. AtMYC1 not only interacts with GL3, but also with many other TFs, both activators and repressors of trichome biogenesis, such as *CPC*, *TRY*, *TTG1*, *GL1*, and *MYB23*, to establish the fate of a cell towards the initiation (or not) of the trichomes [92,93,94]. However, in contrast to GL3 and EGL3, the AtMYC1 protein appears to be unable to form homo- or heterodimers with GL3/EGL3 [92]. In *atmyc1* mutants, the transcription patterns of *GL2*, *TRY*, and *CPC* remain unchanged [95]. In co-expression experiments of the *AtMYC1* gene with *TRY* or *CPC* repressors, both AtMYC1 recruitment in the nucleus and the transport of TRY/CPC TFs from the cytoplasm in the nucleus were observed [95]. Therefore, *AtMYC1* is able to inhibit the role of *TRY/CPC*.

In *Arabidopsis*, *CSN5a*, which encodes the COP9 5a signalosome subunit, is involved in the production of trichomes, as well as in the biosynthesis of various secondary metabolites, such as phenylpropanoids, carotenoids, and zeatin glycosides [96]. A *csn5a* mutant, *sk372*, characterized by enhanced anthocyanin accumulation, also shows a significant reduction in trichome number and abnormal trichome development. In addition, the mutant phenotype is associated with an over-expression of *MYB75* and a down-regulation of *GL2*. The *sk372* mutant also shows a complex modulation of the transcription of members of the MBW activator complex [96].

In *Arabidopsis, MADS* box genes also appear to control trichome development. *AGAMOUS* (*AG*) negatively interferes with the branching process of trichomes differentiated on carpel valves [97]. *AG* represses the differentiation of trichomes in the carpels by controlling the response to cytokinins and interacting with the *KANADI1* gene [98].

In epidermal cells, the *TOO MANY MOUTH* (*TMM*) gene was initially implicated in the control of stomata distribution and patterning [6]. However, in *Arabidopsis* plants over-expressing *TMM,* the trichome density on leaves is significantly reduced, and most of the trichomes display abnormal ramifications [99]. The phenotype of these transgenic plants resembles in some respects that of the *gl1* and *ttg1* mutants, with few or no trichomes, as well as *stichel*, *angustifolia*, and *zwichel* mutants, with trichomes with a reduced number of branches [1,100,101]. The reduced number of trichomes is more evident in organs of the reproductive phase than in leaves or stems during the vegetative phase. These data suggest that *TMM* performs a more important function in advanced stages of plant development [99].

The MBW activator complex controlling trichome initiation also positively controls the genes that act in later stages of the *Arabidopsis* flavonoid biosynthetic pathway, including *MYB75/90/113/114*, *GL3/EGL3/TT8*, and *TTG1* [44,49,93,102,103,104]. Conversely, *TRY* and *CPC* repressors compete with R2R3-MYB TFs for binding bHLH factors, altering the MBW complex; therefore, the differentiation of trichomes, as well as key steps of anthocyanin biosynthesis, is repressed at the same time [105,106].

In eukaryotes, including plants, the ubiquitin/26S proteasome system (UPS) plays an essential role for protein turnover [107,108]. The conjugation of ubiquitin to proteolytic substrates is needed to mark them for degradation. The 26S proteasome, a multisubunit ATP-dependent protease complex, consists of two functionally distinct complexes, the 20S core protease (CP) and the 19S regulatory particle (RP) [108,109]. Patra et al. [107] demonstrated that UPS post-translationally regulates the MBW activator complex, showing that both GL3 and EGL3 are unsteady and marked for the UPS-dependent proteasome degradation. The UPS includes E1, E2, and E3 enzymes, whose combined action is responsible for the conjugation of polyubiquitin chains, which target proteins for proteolysis, mediated by the 26S proteasome [110,111,112,113]. The E3 ubiquitin-protein ligase (UPL3) mediates the proteasomal degradation of both GL3 and EGL3 [107]. In addition, mutation in the *gl3* locus negatively perturbs *UPL3* transcription, but over-expression of *GL3* up-regulates it. These results suggest the probable existence of a regulation cycle concerning *GL3* and *UPL3* [107].

## 3. Gene and Hormonal Interaction in Trichome Development

Phytohormones regulate trichome differentiation, but the ways in which they act are not fully known. The cytokinins (CKs) stimulate the formation of the trichomes overall on the inflorescences, while the gibberellins (GAs) and jasmonic acid (JA) act synergistically on the induction and number of hairs on various organs [5,9,114,115,116]. Therefore, the three phytohormones act positively on regulation of trichome growth; by contrast, the salicylic acid (SA) induces a repressive control on hair initiation [117]. Gibberellins regulate a number of developmental processes in plants, including seed germination, cell elongation, and flowering. Much evidence demonstrates that GAs also control some aspects of trichome morphogenesis. Exogenous treatments with GA on the hairless mutant, deficient in GAs, *gal-3*, stimulate the formation of trichomes, suggesting positive action from GAs on the growth of leaf hairs [118]. GAs promote the development of leaf hairs in *gl1* mutants by directly regulating the *GL1* gene [119]. Moreover, GAs regulate the transcription levels of *GL3*, *TTG1*, and *TRY* [9]. The *Arabidopsis* DELLA proteins are inhibitors of GA signaling and encoded by a family of five genes: *GIBBERELLIC ACID INSENSITIVE* (*GAI*), *REPRESSOR OF ga1-3* (*RGA*), and three *RGA-LIKE* genes (*RGL1*, *RGL2,* and *RGL3*). Among the five DELLA proteins, RGA and GAI play key roles in trichome formation. Mutations in *RGA* and *GAI* restore trichome initiation in the *ga1-3* mutant. Furthermore, GAs exert a positive effect on the transcription level of *GL3*, *TTG1*, and *TRY*. CKs and GAs also activate the expression of genes coding for “zinc-finger” C2H2 TFs, such as *GLABROUS INFLORESCENCE STEMS* (*GIS*), *GIS2*, and *ZINC FINGER PROTEIN 8* (*ZFP8*) (Figure 1), which are supposed to control in concert the transcription of *GL1* and *SIM* [120,121]. In the control of hair development, another C2H2 protein, ZFP6, seems to function as an integrative hub of GA and CK signals in promoting trichome formation in *Arabidopsis*. *ZFP6* expression is induced in response to GA and CK treatments [5]. *ZFP6* can activate the expression of *ZFP5*, and then *ZFP5* promotes *GIS*, *GIS2,* and *ZFP8* expression [122]. Furthermore, *C2H2* members (i.e., *GIS2* and *ZFP8*) increase the transcription of *GL1* and *GL3* (Figure 1). Therefore, *GIS*, acting upstream of the MBW activator complex, promotes trichomatous lining because of hormone action [120,123,124,125,126,127]. The *SPINDLY* (*SPY*) gene inhibits the GA signal [128,129], and *spy* mutants display an excessive number of trichomes. Gan et al. [123] showed the hierarchical relationship of *GIS* with respect to *GL1* and *SPY*. The same authors demonstrated that *GIS* is in contrast with the action carried out by the repressor gene *GAI* [123]. Recently, further TFs belonging to the GIS clade and their redundant functions in GA and CK signaling have been identified [124,125,127]. *Arabidopsis* mutants, defective in GIS-clade function, show a low hair density in both leaves and flower, whereas high concentration of these proteins generates a promotive effect in hairs development [123,124,125,127,130]. In *Nicotiana benthamiana*, *NbGIS* is essential for inducing glandular outgrowths with the concomitant presence of the GA signal [131].

In *Arabidopsis*, the TRICHOME-RELATED PROTEIN (TRP) is a recently isolated TF with an inhibitory effect on hair development in the presence of GAs [132]. The *trp* mutant has more hairs on vegetative and reproductive organs in comparison to normal plants. By contrast, plants over-expressing *TRP* exhibit fewer outgrowths due to GA treatments. A hierarchical relationship between *TRP* with the *ZFP5* and *ZFP8* genes has been proposed [132].

TEMPRANILLO1 (TEM1) and TEM2 TFs, initially detected as inhibitors of flower induction, belong to the small plant-specific RELATED TO ABI3 AND VP1 (RAV) family [133,134]. TEM1 and TEM2 also suppress trichomatous coating through the regulation of GA synthesis and localization in the leaf mesophyll [135]. TEM1 and TEM2 operate, not exclusively, with respect other factors, to suppress key genes required for trichome initiation and growth [135]. Since *tem2*-*2* mutant plants produce more trichomes than *tem1-1* and normal plants, *TEM2* represents a more essential gene in trichome initiation in comparison to *TEM1*.

In *Arabidopsis*, a subunit of the ubiquitin-mediated 26 S proteasome (RPN1a), involved in the development of branched trichomes, interacts with both GAs and CKs [5]. Mutations in the *RPN1a* locus generate more branched trichomes on leaves [136]. In the *rpn1a* mutant plants, the transcription levels of *ZFP5*, *ZFP6*, *GIS*, *GL1*, *GL2*, *GL3*, *TTG1*, and *MYB23*, positive elements of hair development, are up-regulated. In addition, the activity of *FURCA4* (*FRC4*), which is important for inducing trichome branching, is also enhanced in the *rpn1a* mutant in comparison to wild type [122,136]. The mRNA level of *RPN1a* is highly inhibited by GA and CK treatments. *RPN1a* is likely involved in trichome development through the GA and CK signaling pathways [136].

6-benzylaminopurine (BAP, CK) induces hair development, since *Arabidopsis* BAP-treated plants develop a high density of hairs on their leaves. Nevertheless, outgrowths are not elongated and have a lower DNA content than in untreated plants. These data suggest that BAP plays a negative role in the endoreduplication cycle (see the next paragraph). Moreover, exogenous treatments with BAP stimulate the gene expression of *GL1*, *MYB23*, *GL3*, and *EGL3* [137]. On the other hand, CKs also increase trichome formation during the reproductive stage; in fact, this class of hormones promotes the trichome complement in the inflorescence stems [115].

Trawl and Bergeson [138] first showed that wounds and JA significantly promote trichome development. JA is involved in trichome differentiation by reducing Jasmonate ZIM-domain (JAZ) proteins, as well as eliminating the interactions between JAZ with both bHLH and MYB factors, to promote the expression of hair activators [9,139] (Figure 1). A molecular mechanism underlying the GA-JA synergy in trichome development proposes that both JAZ and RGA bind the regulators of trichome development, GL3, EGL3, and GL1 [5]. GA and/or JA signals control the level of these repressor proteins via 26S proteasome-dependent proteolysis and maintain the stable transcription of the activators that induce trichome formation.

JA and SA enhance the resistance of plants to pathogens and insect pest attacks, but in *Arabidopsis*, they are also involved in the formation of the trichome complement [132,140]. JA is inductive in outgrowths initiation on the leaf, as well as on the accumulation of anthocyanins. However, mutants deficient in JA can differentiate trichomes [139]; therefore, JA appears to be not crucial for their development. It is likely that the influence of JA on trichome development could be species-specific or linked to the trichome typology.

In *Artemisia annua*, a key protein to induce the growth of different types of trichomes is the HOMEODOMAIN PROTEIN 1 (AaHD1), an HD-ZIP TF, whose role is likely JA-mediated [141]. Notably, in *A. annua*, the production artemisinin, a useful molecule against the *Plasmodium* causal agent of malaria [142], depends on trichome development. TRICHOME AND ARTEMISININ REGULATOR 1 (TAR1), an APETALA2 TF, plays important roles in regulating both trichome development and artemisinin biosynthesis [143]. Notably, Tan et al. [143] identified some specific targets of the TAR1 protein as *AMORPHA-4, 11-DIENE SYNTHASE* (*ADS*) and *CYTOCHROME P450 MONOOXYGENASE* (*CYP71AV1*) genes.

JA, CKs, and GAs promote hair initiation in *Arabidopsis*, but their roles can diverge with respect to hair maturation [114]. Furthermore, these hormones exert their promotive effect on initiation with analogies across different lineages, despite the involvement of unrelated regulatory networks [114].

SA has an opposite effect with respect to GAs, CKs, and JA, reducing the density of trichomes in *Arabidopsis* leaves and inactivating the effects of JA [138]. However, the last relationship has not been observed in other species [144]. The recessive *constitutive expresser of PR gene5* (*cpr5*) mutant of *Arabidopsis* shows a phenotype which has been severely dwarfed. The *cpr5* mutant has a higher content of SA and sugar-conjugated SA in comparison to wild type. Interestingly, the trichome complement of *cpr5* leaves shows a significant reduction [145]. *cpr5* plants also display leaf trichomes of reduced size and decreased ramification. Furthermore, these outgrowths show a reduced birefringence, which suggests an altered composition of cell wall structure [146]. The loss of function of *CRP5* activity has consequences on trichome size and nuclear DNA content, and these aspects can be epistatic to the effects of mutations at the *TRY* locus or over-expression of *GL3* [146].

Notably, in some species, prickles (deterrent structures against herbivore and insects) are the extensions or modifications of glandular trichomes. The Trihelix Transcription factor GT2-Like 1 (GTL1), a main regulator of ploidy-dependent trichome growth and drought tolerance, enhances the expression of defense genes and, at the same time, hinders other key players with roles in trichome morphogenesis. In this contest, it is interesting that GTL1 coordinates different genetic elements implicated in various aspects of SA metabolism [147]. Recent data, collected by a differential transcriptomic analysis of epidermis of both *prickly* and *prickleless* mutants of *Solanum viarum* (an important medicinal plant), show that the activity of several defense regulators, such as ethylene, SA, and PATHOGEN RELATED-proteins, is significantly repressed in *prickleless* mutants [148].

Ethylene governs the development of leaves, flowers, and fruits. It may promote, inhibit, or induce senescence, depending upon the optimal or sub-optimal ethylene levels. Ethylene also manifests its effects on the complexity of the trichome, acting negatively on the branching process. In fact, mutants with low levels of ethylene develop only simple trichomes [149]. It was suggested that an ethylene receptor gene, *ETHYLENE RECEPTOR 2* (*ETR2*), could influence the microtubule formation of the cell cytoskeleton by acting upstream of both *CHROMATIN ASSEMBLY FACTOR1* (*CAF1*) and *TRY*, and its function appears strictly dependent on both *GL2* and *GL3* activity [149].

Brassinosteroids (BRs) are steroid hormones essential for plant growth and development, controlling the division, elongation, and differentiation of various cell types throughout the entire plant life cycle. BRs are also involved in trichome development. The *Arabidopsis brassinosteroid, light and sugar1* (*bls1*) mutant, defective in BR response, shows a reduced hair proliferation on leaf epidermis [150]. In addition, this mutant displays a pleiotropic phenotype: short hypocotyl, expanded cotyledons, short roots, compact leaf rosette, reduced height, delayed bolting, and hypersensitivity to metabolized sugars [150].

In tomato, trichome development in different mutants suggest that hormones such as ethylene, GA, and auxin can indirectly modify the trichomatous coating of a specific genotype, for their effects regarding epidermal cell area on leaves [151]. For example, the *epinastic* (*epi*) mutant characterized by high ethylene content develops a lower density of hairs in comparison to wild type, but this trait can be related to an increase in epidermal cell surface [143]. Nevertheless, hormones such as BRs and JA can directly influence hair density. In particular, the (*dumpy*) *dpy* mutant (BR-deficient) shows enhanced pubescence [152], while the *jasmonic acid insensitive1-1* (*jai1-1*) mutant displays an opposite phenotype [151,153].

## 4. Regulation of the Cell Cycle and Trichome Complexity

Many plants produce multicellular and/or branched trichomes, through three phases: (i) initiation with the induction of cell competence, (ii) endoreduplication, (iii) expansion and morphogenesis. After the determination of the cell fate, the progenitor cells of the trichomes stop the mitotic cycle to move into the endoreduplication phase—a cellular condition in which the duplication of the chromosomes in phase S (DNA synthesis) of the interphase does not follow the entry into the mitotic cycle to form two daughter cells with normal DNA content. Therefore, the cell will be endoreduplicated. With the entry into mitosis of the endoreduplicate cells, polyploid cells can be generated [154].

The endoreduplication event in trichome precursor cells is the basis of the branching and expansion processes that underlie the trichome complexity. Cells that have acquired the competence to become multicellular trichomes initially elongate and, subsequently, divide perpendicular to the epidermal plane, in a condition of continuous cell division [155]. Analogously, the number of ramifications depends on the cellular content in DNA: more ramifications are found if there is a high level of endoreduplication cycles, while the complexity of the hairs decreases when the levels of endopolyploidy are reduced. Cell cycle control also plays an important role in the early developmental stages of trichome initiation [7,9,156].

**Figure 2:**
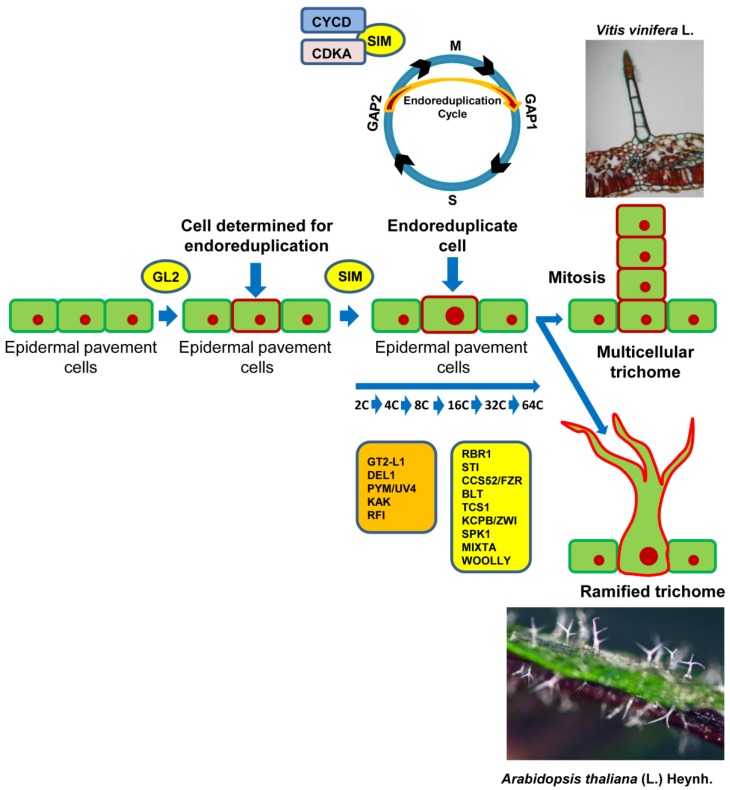
A simplified model for the differentiation of multicellular and ramified trichomes. An epidermal pavement cell activated by *GLABRA2* (*GL2*) becomes determined for trichome initiation (red cell wall). The *SIAMESE* (*SIM*) gene promotes the endoreduplication cycle (see also Figure 1). SIM interacts with D-type cyclin-CYCLIN-DEPENDENT KINASE A (CYCD-CDKA) complexes, which normally operate at the G1/S and G2/M transitions, to suppress entry into phase M. The nuclear DNA content increases from 2C to 64C. The endoreduplicate cell can follow two fates, also in relation to the species: entering the mitosis process, originating a multicellular trichome as in the grape (*Vitis vinifera* L.), or promoting the development of branched trichomes, as in *Arabidopsis thaliana* (L.) Heynh. In the orange box are indicated some negative regulators involved in the development of complex trichomes: *GT2-LIKE1 trihelix* (*GT2-L1*), *DP-E2F-Like1* (*DEL1*), *KAKTUS* (*KAK*), *POLYCHOME//UV-INSENSITIVE4* (*PYM/UVI4*), and *RASTAFARI* (*RFI*). By contrast, in the yellow box are indicated some positive regulators: *RETINOBLASTOMA RELATED1* (*RBR1*), *STICHEL* (*STI*), *CELL CYCLE SWITCH 52A2/FIZZY-RELATED1* (*CCS52A2*)/*FZR1*, *CCS52A1*/*FZR2*, *BRACHLESS TRICHOME* (*BLT*), *TRICHOME CELL SHAPE 1* (*TCS1*), *KINESIN-LIKE CALMODULIN-BINDING PROTEIN*/*ZWICHEL* (*KCBP/ZWI*), *BRACHLESS TRICHOME* (*BLT*), *SPIKE1* (*SPK1*), *MIXTA* and *WOOLLY* (*WO*). (See the text for the specific activity of each gene on cell cycle regulation). G1: Gap 1; G2: Gap2; M: mitosis, S: DNA synthesis.

In *Arabidopsis*, the trichomes show three branches, originated by a phase that includes four endoreduplication cycles. The trichome branching is coordinated by regulatory genes that have different roles in controlling how a cell begins to perform its endoreduplication cycles (Figure 2). Among others, the genes *GL3*, *TRY*, *RBR1*, *CELL CYCLE SWITCH 52A2/FIZZY-RELATED1* (*CCS52A2*)/*FZR1*, *CCS52A1*/*FZR2*, *SIM*, *STICHEL* (*STI*), *KAKTUS* (*KAK*), *POLYCHOME*/*/UV-INSENSITIVE4* (*PYM/UVI4*), and *RASTAFARI* (*RFI*) play a crucial role in the endoreduplication process [9,88,157,158,159]. Therefore, both *GL3* and *TRY* genes, in addition to possessing a main function in the initiation of trichomes (Figure 1), also participate in the regulation of branching [160]. The *Arabidopsis gl3* mutants produce trichomes with reduced ramifications due to fewer cycles of endoreduplication, compared to *try* mutants, characterized by additional endoreduplication cycles and a high number of ramifications [160].

RBR and E2F/DP TFs have a prominent role in the pathway of cell cycle regulation [161]. In the *Arabidopsis* genome, there is a single copy of the *RBR* gene, while the E2F/DP proteins represent a more complex TF family [162]. In *Arabidopsis*, three E2F (a, b, and c) proteins that possess a typical organization of conserved motifs, including one N-terminally located DNA-binding domain (DBD), DP heterodimerization, transactivation, and RBR-binding domains, have been identified [163]. These proteins heterodimerize with one of the two DP proteins (a and b), giving rise to functional TFs [164]. Kosugi and Ohashi [165] showed that E2Fa/DPa heterodimers operate essentially as transcriptional activators and regulate both the cell and the endoreduplication cycle. Desvoyes et al. [157] demonstrated that RBR restricts cell division during early leaf development. In the subsequent phases, once the transition to the endoreduplication program has taken place, RBR mainly limits the progression of the normal cell cycle through extra endoreduplication rounds. Therefore, in *Arabidopsis*, RBR-mediated regulation of the endoreduplication cycle is strictly dependent on the growth stage of leaf development [157].

Both *CCS52A1* and *CCS52A2* are key players that promote the shift from the cell cycle to the endoreduplication cycle. The E2FA–RBR complex transcriptionally represses the expression of both *CCS52A1* and *CCS52A2*. In addition, the *GT2-LIKE1* TF negatively regulates *CCS52A1* transcription [166], whereas the CK-activated *ARABIDOPSIS RESPONSE REGULATOR2* activates its transcription [167]. Furthermore, in mitotically dividing cells, *CCS52A2* transcription is suppressed by E2F and DP-E2F-Like 1 (DEL1) TFs, which operate as a negative regulators at the beginning of the endoreduplication cycle [158,168]. *FZR2* controls the induction of the initial cycles of endoreduplication, while the remaining cycles are regulated by *FZR1* and *FZR3*. In *Arabidopsis*, most, but not all, endoreduplications are mediated by the expression of *FZR1* and *FZR2*. However, Larson-Rabin et al. [159] showed that the reduced *FZR2* activity decreases both the number of endoreduplication cycles and the expansion of the trichome. By contrast, an over-expression of *FZR2* is enough to allow extra cycles of endoreduplication in epidermal cells of leaves, roots, and flowers, leading to an alteration of the trichome size [159].

Cell cycle progression in leaf trichome differentiation is controlled by some CDKs, a family of protein-serine/threonine enzymes. CDKs are regulated by cyclin-dependent kinase inhibitors (CKIs). Plant genomes encode two distinct families of CKIs, the INHIBITOR/INTERACTOR OF CDC2 KINASE/KIP-RELATED PROTEIN (ICK/KRPs) family and the SIAMESE-RELATED (SMR) family, in which SIM is included [169]. CDKs, along with their regulatory subunit cyclins (CYCs), play their roles in different phases of the cell cycle [170,171] (Figure 2). The D-type cyclin-CYCLIN-DEPENDENT KINASE A (CYCD-CDKA) complexes are active in the phase of the GAP1 (G1)/S and G2/Mitosis (M) transitions, while the CYCA/B-CDKA/B complexes are operative at the time of the G2/M transition [169,170,171].

Two genes fundamental to regulating the endoreduplication cycle are *SIM* and *STI* [172]. *SIM* acts together with D-type cyclins (CYCDs) and CDKA to suppress entry into the M phase (Figure 2). Therefore, the normal cell cycle is replaced by the endoreduplication cycle [173]. In *Arabidopsis sim* mutants, the trichomes divide during development and become multicellular, but in reduced numbers per leaf [159,174,175]. The cyclins also play a role in the specification of the substrate of the cyclin–CDK complex. In fact, only specific “cyclin–CDK pools” promote the initiation of DNA replication, through the phosphorylation of the specific substrate for the transition, in this instance, from the G1/S or G2/M phases [176]. The transcription of the *CYCLIN B1*; *2* gene encoding a type B cyclin, which regulates the G2/M transition, is usually inhibited by the *SIM* gene [177]. Really, the ectopic expression of *CYCLINB1; 2* stimulates the differentiation of branched trichomes in both *Arabidopsis* wild type and *sim* mutant plants. The *CYCLIND3; 1* gene is specific to the formation of type D cyclin that, in *Arabidopsis* trichomes, induces cell division [178,179]. Schnittger et al. [177] showed that the activation of ICK/KRP, an inhibitor of CDK that interacts with CYCD, in trichomatous cells rescues the *sim* mutant phenotype.

The regulatory control of the cell cycle in the initiation of complex unicellular trichomes differs substantially from that related to cellular determination addressed to the development of multicellular trichomes. However, in both cases, the transition from the mitotic phase to the endoreduplication cycle is fundamental. In *Arabidopsis*, mitosis is definitively inhibited and replaced by the subsequent endoreduplication phase, allowing the establishment of cellular competence directed towards the development of branched unicellular trichomes. In tomato and other species with multicellular hairs, mitosis is inhibited and, similar to the previous model, the endoreduplication phase is triggered; however, in the determination of cell fate, the pre-trichomatous cells will resume some mitotic cycles, allowing the formation of multicellular trichomes [180].

By increasing the mitotic process, the development of multicellular trichomes is favored, with respect to the initiation and activation of the trichomes themselves. The inhibition of the initiation of the trichomes in *Arabidopsis* plants, characterized by an increase in the mitotic cycle, is due to the inability of the activator complex to reach a threshold level capable of promoting the determination of leaf follicles [181]. *STI* appears to make a key function on the differentiation of secondary branches. The *STI* gene encodes a protein characterized by a motif, with a high similarity to the gamma eubacterial DNA-polymerase III subunits that bind ATP [172]. The N terminal region of the *STI* product also contains two PEST domains, while two nuclear localization signals (NLS) are placed within the N terminal and the C terminal region, respectively [182]. Xi et al. [182] suggested that in *Arabidopsis* the PEST domain is fundamental for the functioning of the *STI* gene in the regulation of the process underlying the proper development of branched trichomes. This is deduced by the direct interaction of *STI* with the *BRACHLESS TRICHOME* (*BLT*) gene, which plays a key role in the cellular form and in the control of the endoreduplication cycle [182,183]. Although *blt* mutants maintain unmodified trichome DNA content, *BLT* over-expression contributes to determining a further endoreduplication cycle [183]. Furthermore, loss-of-function mutations for *BLT* increase the number of complex trichomes in *sim* mutant plants [183].

In *Arabidopsis*, Perazza et al. [184] isolated five mutants—*polychome* (*pym*), *rastafary* (*rfi*), *kaktus2* (*kak2*), *kak3,* and *kak4*—showing leaf trichomes with an increased branching phenotype (five–six branches) in comparison to wild type. The phenotype of these mutants strongly resembles both *try* and *spy* mutant plants. Furthermore, trichomes of *pym*, *spy-5*, *kak*, and *rfi* mutant plants show a significant increase in the content of nuclear DNA, giving new support for a link between the endoreduplication cycle and trichome complexity [184]. *KAK*, *PYM,* and *RFI* specifically repress the endoreduplication cycle in trichomes. The *KAK* gene encodes a protein that belongs to a monophyletic subgroup of HECT proteins, which also includes armadillo-like repeats (repeated sequences of amino acids of about 40 residues in length) [185]. Downes et al. [186] recognized a family of seven ubiquitin-protein ligases containing the HECT motif (UPL1-UPL7). The *upl3* mutant plants show an altered development of the trichomes with five or even a greater number of branches, in comparison to the three branches of wild type trichomes. Cells determined for trichome initiation in *upl3* mutants often enter in additional endoreduplication cycles, with a consequent increase in nuclear DNA content up to 64C. Genetic analysis demonstrates allelism between the *upl3* and *kak-2* mutations. In addition, Downes et al. [186] showed that the *KAK* gene represses the endoreduplication cycle, through the degradation of a specific protein, characterized by a ubiquitous system.

The *PYM/UV-INSENSITIVE4* (*UVI4*) gene is a fundamental negative regulator necessary for the correct mitotic progression in the cell cycle and the maintenance of meristematic cellular competence inhibiting the cellular differentiation [187]. Both *uvi4* and *del1*-*1* mutant plants display a significant increase in trichome branching in comparison to wild type [188]. A quantification of the nuclear size of trichomes revealed an increase in the DNA content in trichome cells of *uvi4* mutant plants, similar to the one found in the *del1-1* trichomes. This evidence confirms the role of the *DEL1* gene in the suppression of the transition from the mitotic phase to the entry in the endoreduplication cycle [188]. The double *uvi4*; *del1*-*1* mutant shows highly branched trichomes in comparison with single mutants. This phenotype is closely related to a corresponding increase in the DNA content and the nuclear size of trichomatous cells [188].

Much evidence shows that the structural organization and dynamics of cortical microtubules (cMTs) are strongly interconnected with the complexity of both branched and multicellular trichomes [189,190,191,192]. cMTs show high flexibility as a function of their intrinsic ability to pass rapidly from elongation to shortening conditions. These dynamic processes are regulated by factors interrelated with cMTs and by the concentrations in the matrix of α/β-tubulin heterodimers, which are indispensable for their formation [193,194]. In trichome development, the dynamic structure of cMTs drastically changes near the branch point [190]. Furthermore, mutations in genes involved in the proper establishment of α/β-tubulin heterodimers or in modifications of cMTs dynamics frequently determine the development of abnormal trichomes, with profound changes in the branching growth [189,191,192,195,196,197]. Abe et al. [192] analyzed the semi-dominant *lefty1* and *lefty2* mutants of *Arabidopsis* derived from two gene mutations, which lead to an amino acid substitution in both α-tubulin 6 (TUA6) and α-tubulin 4 (TUA4) proteins. The seedlings of the double mutant show peculiar phenotypes with a helical growth in the hypocotyls, together with a radial cell expansion in the root elongation zone. The *lefty* double mutants display an abnormal organization of cMTs and decreased trichome branching [192]. Notably, Abe and Hashimoto [196] demonstrated that if a sequence encoding for the hemagglutinin (HA) epitope is linked to the N-terminal region of the TUA6 protein and constitutively expressed, *Arabidopsis* plants display a semi-dwarf phenotype with poor fertility. Plants over-expressing the HA-TUA6 protein also show cMTs more prone to polymerization and the development of highly branched trichomes [196].

The *TRICHOME CELL SHAPE1* (*TCS1*) gene encodes a protein characterized by a coil–coil motif which binds cMTs and promotes their proper organization. TCS1 is physically associated with the kinesin-like calmodulin-binding protein/ZWICHEL (KCBP/ZWI), a cMT system implicated in the regulatory pathway that controls the number of branches [198]. Chen et al. [199] performed genetic analyses in *Arabidopsis* mutants, demonstrating that *kcbp/zwi* is epistatic to *tcs1* with regard to the number of branches in leaf trichomes. Therefore, it is likely that TCS1 can interact with KCBP in the control of trichome shape by influencing the stability of the cMT system [199].

Recently, Liang et al. [200] identified an *Arabidopsis* mutant, *aberrantly branched trichome1-1* (*abt1-1*), characterized by a phenotype which displays a reduction in trichome complexity. The *abt1-1* mutation is allelic to the *SPIKE1* (*SPK1*) gene, which encodes a member of the CDM family of proteins, which plays an essential role as a guanine nucleotide exchange factor (GEF) [200]. CDM is the acronym for the genes *CED-5* from *Caenorhabditis elegans*, *DOCK180* from humans, and *myoblast city* for *Drosophila melanogaster* [201,202]. *SPK1* plays a key role in the arrangement of nuclei in cells during the initiation and development of trichomes. In addition, the regulatory and coordinated action that underlies the branching of trichomes is linked to the interaction between *SPK1*, *ANGUSTIFOLIA* (*AN*), and *ZWI* genes [200].

The *MIXTA* gene codifies for a TF R2R3-MYB that controls the determination of cells originating from multicellular trichomes on petals of *Antirrhinum majus* [203]. The over-expression of two *MIXTA* genes, *MYB MIXTA LIKE1* (*AmMYBML1*) and *CotMYBA,* of *A. majus* and cotton, respectively, promotes the formation of multicellular trichomes in *Nicotiana tabacum* [9,43,203]. These results suggest that a *MIXTA-LIKE* gene can actively operate in the formation of multicellular trichomes in several different species [204,205]. Indeed, these R2R3-MYB TFs conserve the DNA binding domain, like GL1, and it is likely that in some species the activity of *MIXTA-LIKE* genes replaces the role played by *GL1* in *Arabidopsis* [9]. In fact, *GL1* over-expression in tobacco appears to have no effect on the production of trichomes, and the ectopic expression of *MIXTA* on *Arabidopsis gl1-1* mutants does not generate trichomatous phenotypes [43].

In tobacco, Serna and Martin [206] hypothesized that MIXTA proteins do not require the interaction of *GL3* and *EGL3* to control trichome differentiation, and therefore the MBW complex does not appear to be fundamental as in *Arabidopsis*. Tomato is a complex case, because this species differentiates eight morphologically distinct classes of multicellular trichomes [20,207], including two classes of non-glandular trichomes (III and V) and four glandular classes (I, IV, VI and VII), which have been morphologically characterized [18]. For example, Xu et al. [208] showed that SlMYC1, a bHLH TF, is fundamental for the correct differentiation of class VI glandular trichomes. Notably, in the class VI glandular trichomes of tomato leaves and stems, *SlMYC1* plays an important role also in the regulation of the biosynthesis of mono- and sesquiterpene compounds. In tomato, each type of trichomes follows different regulatory pathways. However, Yang et al. [207] demonstrated that the *WOOLLY* (*WO*) gene fundamentally controls all trichome types. As with the *MIXTA* gene, in *Arabidopsis*, the over-expression of *WO* appears to have no effect on the initiation or development of branched trichomes. These data demonstrate that multicellular trichomes of tobacco and tomato and unicellular trichomes of *Arabidopsis* and cotton are not homologous structures and different gene regulatory networks likely control their developmental pathways [206,207,208,209,210]. It is likely that during evolution, genes such as *WO* and *PROTODERMAL FACTOR2* (*PDF2*) have acquired various biological functions among angiosperms [207]. *Arabidopsis* and cotton are Rosids, while tobacco and snapdragon are Asterids. Conceivably, in Rosids and Asterids, the regulatory pathways controlling trichome development evolved, at least partly, differently at the time of their ancestral separation [206,207,208,209,210].

It has not yet been elucidated whether and how far phytohormones can be involved in the development of multicellular trichomes; however, it seems that JA participates in their initiation, while CKs and GAs promote the outgrowth of multicellular hairs in tomatoes [114]. GA signaling affects trichome branching and endoreduplication. For example, *spy-5* mutants have multi-branched trichomes with a DNA content of 64C. Conversely, *ga1-3* mutants are nearly glabrous and rarely have bifurcated trichomes. GA signaling likely controls endoreduplication cycles via the regulation of *GL1* or its homolog [3]. In addition, it is now known that each hormone class controls the different types of trichomes present in the same species in specific ways. In tomato plants, JA is involved in the preferential activation of type VI trichomes, while CKs activate the development of type VII trichomes [114]. In tomato, auxin also appears to play a key basic function for the proper differentiation of glandular trichomes. *AUXIN RESPONSE FACTOR* (*ARF*) genes encode a large family of proteins involved in hormone signal transduction [211]. In tomato, Zhang et al. [212] identified the *SlARF3* gene, which encodes a protein containing two highly conserved domains, B3 and ARF, but lacks the Aux/IAA motif. A down-regulation of *SlARF3* induces a reduced number of epidermal pavement cells, as well as a decreased density of several classes (I, V, and VI) of tomato leaf trichomes. These results suggest the fundamental function of the *SlARF3* gene activity in establishing the cellular competence for trichome initiation and development in tomato [212].

## 5. Epigenetic Factors Involved in Trichome Development

In developmental biology, gene expression must be regulated at the cellular level and the transcriptional activity must take place respecting specific temporal windows. These aspects require a genetic control that resides in DNA sequences, but in addition, another level of control involves an epigenetic regulation, such as the modification of gene expression in the absence of irreversible changes in DNA sequences. Therefore, it is not surprising that in plants, over 130 genes encoding proteins with roles in epigenetic regulation in plants have been identified [213]. These include: (i) regulator proteins of DNA modification (i.e., DNA methyltransferases, cytosine demethylation and DNA glycosylases, methylcytosine-binding proteins, and proteins required for methyl group donor synthesis); (ii) histone-modifying enzymes and histone variants (i.e., histone deacetylases and histone acetyltransferases, histone methyltransferases and histone demethylases, histone variants, linker histones, and no histone proteins); (iii) polycomb proteins and interacting components; (iv) nucleosome-organizing proteins (i.e., chromatin-remodeling complexes and chromatin assembly factors); (v) small interfering RNAs (siRNAs)- and micro RNAs (miRNAs)-mediated post-transcriptional gene silencing (PTGS) [213,214].

The development of trichomes appears to be influenced by epigenetic modifications. Basic data have been collected analyzing trichome architecture in recessive mutants of *Arabidopsis* related to the trimeric protein CHROMATIN ASSEMBLY FACTOR1 (CAF1) [215,216]. CAF-1 has three subunits and it was originally investigated in human cells [217]. This protein is implicated in chromatin assembly during DNA replication and DNA repair in vivo [218,219,220]. In *Arabidopsis*, genetic defects in CAF1 subunits (i.e., *FASCIATA1* (*FAS1*) or *FAS2*) have been identified in mutants characterized by a different pattern of hairs branching. These mutations involve the activity of the *STI* gene but the mutual relationship is not dependent on the entry into the endoreduplication process [216]. Moreover, Exner et al. [216] suggested that CAF-1 is important for branching outgrowths, without a concomitant role of the *KAK* gene. Endoreduplication in WT hairs is independent from *CAF-1* activity, but it is notable that the same protein is required for the extra series of endoreduplication cycles identified in *kak* mutants. When the function of *CAF-1* is lost, the *H3.2* gene transcription is deregulated. This effect can justify the high content of the H3.2 variant histone in the chromatin of *CAF-1* mutant trichomes [216]. Therefore, in *CAF-1* mutants, the increased trichome ramification is not related to an abnormal endoreduplication phase. It is likely that the two processes act independently [215].

In all organisms, the developmental processes require a dynamic organization of chromatin assembly to modulate complex patterns of gene expression in time and space [221]. A fundamental rule requires the covalent modification of histone proteins by means of acetyl groups, usually in the N-terminal domain of the histones [222]. Between the GENERAL CONTROL NON-REPRESSED PROTEIN5 (GCN5) protein, a histone acetyltransferase, and the transcriptional adaptor protein ADA2 exists a direct interaction; if ADA2 is missing, a deficiency of acetylation is detected [223]. Both the *GCN5* and *ADA2* genes play a critical role in regulating metazoan growth and development. A recent investigation demonstrated that GCN5 acts in the control of trichome initiation and outgrowths through the variation of transcription activities of specific genes by means of H3K9/14 acetylation [224]. In fact, trichomatous cell density enhances in a genotype of *Arabidopsis* defective of *GCN5* gene function. Interestingly, a transcriptome analysis in *gcn5* mutants demonstrated that the expression profile of relevant genes, such as *CPC*, *GL1*, *GL2*, and *GL3*, is repressed. In addition, analyses of chromatin immunoprecipitation suggest that these genes are the specific targets of GCN5. In agreement, Wang et al. [224] showed that GCN5-mediated H3K14/K9 acetylation levels on the Transcription Start Site (TSS) motifs of the same genes are declined.

In *Arabidopsis*, the proteins GCN5 and ADA2b influence cellular growth and division at the leaf level, but also have a key role in molecularly linking endoreduplication and hair branching [225]. Kotak et al. [225] demonstrated that the ploidy levels in *gcn5* and *ada2b-1* mutants are divergent: a low ploidy level in the first mutant and rise of the same parameter in the second mutant, respectively. A reduced ramification with respect to normal plants characterizes the trichomes of *gcn5* and *ada2b* mutants, while the *gcn5-6* mutant shows increased branching in leaf trichomes. Elongation of the trichome stalk and branches also varies in different mutants, with stalk length having an inverse relationship with branch number. The results of Kotak et al. [225] highlight the role of ADA2b and GCN5 to link nuclear content with cell morphogenesis.

### miRNAs and Trichome Development

MicroRNAs (*miRNAs*) are small, endogenous, non-coding RNAs of 20–22 nucleotides in length, widely ubiquitous in nature [226,227,228]. A fundamental aspect of *miRNAs* is their involvement in the control of gene expression at the post-transcriptional level, with a high affinity to specific targets [5,229,230]. In plant development, *miRNAs* attend several phenomena, including trichome differentiation and growth [5,231]. Some explicative examples can be illustrated. *Mucuna pruriens,* an important species with pharmaceutical applications, shows big unicellular trichomes, which are filled by metabolites. The trichomes differentiate on various parts of the *M. pruriens* plant body, but with a non-uniform distribution on all the organs. Trichome density on the pod is the most likely to preserve the seeds from the predatory activity of many insects and other different animals that can damage them [231]. In *M. pruriens*, *miRNAs* (*Mpr-miRNAs*) that regulate genes active in trichome morphogenesis have been identified [232]. Singh and Dhawan [232] showed that *mpr-miR1513* regulates TRANSPARENT TESTA 1 (TT1) TF, while *mpr-miR2673* acts on the GL3 protein. In addition, both *miRNAs* have pleiotropic effects, regulating *GL1*, *GL2,* and *CPR-5* genes [232].

*Xanthium strumarium* is another species with an evident hair coating, specialized in the production of different metabolites (i.e., xanthanolide) for various finalities [233,234,235]. The biosynthesis of active substances in glandular trichomes has been characterized at the transcriptional level. Fan et al. [236] demonstrated that *miRNAs,* such as *miR6435*, *miR5021,* and *miR1134* show a differential expression and are crucial players in controlling genes involved in the metabolism of terpenoid accumulated in trichomes.

In *Arabidopsis*, the activity of SQUAMOSA PROMOTER BINDING PROTEIN-LIKEs (SPLs) regulates organ development, plant ramification, hormone response, and juvenile-to-adult transition. SPLs are a group of plant-specific TFs that share a highly conserved SBP DNA-binding domain, first identified in a protein that binds to the promoter of the *SQUAMOSA* gene of *Antirrhinum majus* [9] In *Arabidopsis*, SPLs are negative regulators of trichome development in the inflorescence stem and floral organs. SPLs participate in trichome differentiation through interaction with the promoters of *TLC1* and *TRY* genes in a time-dependent control [237,238]. The *SPL* gene family includes 17 members, 10 of which are targeted by *miRNA156*. Transgenic *Arabidopsis* plants constitutively expressing *miRNA156* produce ectopic trichomes on the stem and floral organs. Post-transcriptional regulation of different SPLs necessitates of the *miR156* activity, whose expression is linked to the developmental stage of the plant [239,240,241,242]. There is a negative relationship between *SPLs* activity and hair density in the reproductive stage [238,243]. Remarkably, the activity of *SPLs* also influences the transfer of hair differentiation preferentially from the adaxial to abaxial side of leaves [32,118]. The control of *miR156* to target *SPLs* is operative not only in *Arabidopsis* but also in other species: *Oryza sativa* [244], *Brassica napus* [245], *Panicum virgatum* [246], *Medicago sativa* [247], and *Solanum tuberosum* ssp. *andigena* [248]. In *Arabidopsis*, the *miR156*/*SPLs* system presides over the pigmentation by anthocyanin accumulation in stem portions [249]. In *Medicago sativa*, the *TCL1* gene is down-regulated by *miR156OE* [250]. Zhang et al. [251] identified in *Nicotiana tabacum* three expressed sequence tags (ESTs) encoding *miR156*-targeted *SPLs* (*NtSPL2*, *NtSPL4,* and *NtSPL9*). In *N. tabacum* plants over-expressing *miR156*, scanning electron microscope (SEM) analyses indicate that transgenic leaves produce a reduced number of leaf trichomes in comparison to wild type plants [251]. These results reveal that the over-expression of *miR156* delays the shift to the adult stage of transgenic plants. Therefore, the model that includes the acquisition of cellular competence by epidermal cells, trichome initiation, and the various phases of differentiation of both simple and complex trichomes can be used in the study of the juvenile–adult phase transition for different plant organs. Nevertheless, it is necessary to consider that the morphological and developmental characteristics of the trichomes that discriminate each of these phases are probably species-specific.

In *Arabidopsis*, analyzing the regulatory network that controls the differentiation of trichomes on the stem, Xue et al. [252] showed that some TFs belonging to the large GRAS family, such as LOST MERISTEMS 1 (LOM1), LOM2, and LOM3, targeted by timing *miR171*, act by adjusting the SPL function by direct physical interactions between proteins. It is likely that LOMs induce the differentiation of trichomes by reducing the action of SPL on the inhibition of trichome development. In particular, the activity of LOMs is dependent on SPLs, because they operate as positive regulators of repressor genes of various stages of trichome development, such as *TCL1* and *TRY* [238]. Furthermore, Xue et al. [252] demonstrated that the transcription of the *miR171* gene is modulated by its targeted LOMs, giving rise to a feedback mechanism.

*Gossypium hirsutum*, an allotetraploid species, is the most cultivated cotton in the world. In the genome of this species, two homoeologous genes, *GhMYB2A* and *GhMYB2D,* were identified. Phylogenetic analyses demonstrate the high identity of both *GhMYB2A* and *GhMYB2D* with the *Arabidopsis GL1* gene [253]. In cotton, Xie et al. [254] identified at least seven unique *miRNAs* and eleven *trans-acting siRNA* (*ta-siRNA*) candidate genes, which participate in the trichome regulatory interaction network. Results collected from functional genomics experiments, genetic transformation, and the morphological and molecular characterization of mutants suggest a distinct spatial and temporal activity between the *GhMYB2A* and *GhMYB2D* genes. This functional divergence is modulated by *miR828*-directed *ta-siRNA* activity, which controls both the biogenesis of *Arabidopsis* leaf trichomes and the differentiation of cotton fibers [254].

One of the main products of the different *Mentha* species is an essential oil, which is stored in the glandular trichomes after its biosynthesis [13,255]. *miR5021* participates in the regulation of various TFs, especially some belonging to the large MYB family, and is fundamental in the metabolism of mint essential oils [231]. In mint, *miR156* plays a dual role by regulating the biosynthesis of oils and by controlling the development of trichomes with its activity on TFs of the bHLH family. In *Arabidopsis*, the *MYC* gene is an activator of trichome initiation [92]; in *Mentha*, *MYC* is regulated by *miR5015*. Notably, *miR5015* is also a regulator of WD-rich proteins, fundamental for inducing cellular competence for trichome initiation, cell cycle regulation, and mechanisms of signaling systems that trigger cell responses [256]. Therefore, in mint, the activity of each component of the trimeric MBW activator complex is controlled by distinct *miRNAs*.

During trichome development, *miRNAs* also act in the regulatory pathways of hormone signaling. Mutations at the *SPY* gene locus, a repressor of the GA signal, determine an increase in the density of trichomes in different plant organs [5,118,119]. In mint, *miRNA* families (*miR156* and *miR5015*) regulate the *SPY* gene, whereas *miR5021* modulates ethylene activity, which plays a key role in trichome branching [231]. In *Arabidopsis* and rice, *miR160* controls the auxin response factor (ARF) [257], while in conditions of different types of stresses, *miR414* is fundamental in the regulatory pathway that controls auxin-induced protein (IAA4).

Together, these observations suggest that in different species, various steps of trichomes development (e.g., determination of cellular competence, initiation and growth, and metabolite storage) are largely controlled by regulatory systems, in which various *miRNAs* are implicated.

## 6. Conclusions

The differentiation of trichomes from epidermal pavement cells constitutes an interesting developmental process, which can provide valuable information for understanding cell fate determination and maintenance. Although many trichome-patterning genes have been identified, thanks to the isolation and characterization of a plethora of different mutants, the new findings provide new challenges and require further investigation. The initiation of these highly specialized epidermal protrusions is controlled both spatially and temporally. Therefore, important molecules involved in heterochronic processes can be identified through investigations of the activation/repression of trichome initiation. Trichomes first attracted botanists and were of interest in plant taxonomy, especially, in the past. Actually, these structures are not just a research field in plant ecology and plant protection but represent useful pharmaceutical factories, and in the future, they will inspire non-conventional human applications [258]. The control of developmental processes in trichome differentiation is a very complex issue, where molecular players act in different regulatory networks: several TFs (both activators and/or repressors of initiation and cell cycle), hormones, and epigenetic factors. The knowledge of the genetic network implicated in trichome development has been deepened above all in *Arabidopsis,* exploiting the molecular facilitations allowed using this plant model; however, it is increasingly evident that peculiar aspects of the transcriptional regulation network exist in other species. BAP, GAs, and JA are major phytohormones with roles in trichome development; nevertheless, the relationship between growth regulators and TFs will require further investigation. Analogously, an interesting topic will be to clarify molecular mechanisms that govern the differentiation of glandular trichomes in crops and medicinal species. As pointed out by Pattanaik et al. [5], the genomic database TrichOME (www.planttrichome.org) is a useful source of information for investigating the molecular origin of different trichomes types. Finally, the control of trichome development at the post-transcriptional level and the epigenetic factors involved in this phenomenon are only partially known and future research will be required to obtain a wider perception of the very complex network of different players governing the trichomatous complement in plants.
